# On-chip terahertz isolator with ultrahigh isolation ratios

**DOI:** 10.1038/s41467-021-25881-0

**Published:** 2021-09-22

**Authors:** Shixing Yuan, Liao Chen, Ziwei Wang, Wentao Deng, Zhibo Hou, Chi Zhang, Yu Yu, Xiaojun Wu, Xinliang Zhang

**Affiliations:** 1 Wuhan National Laboratory for Optoelectronics and School of Optical and Electronic Information, Huazhong University of Science and Technology, 430074 Wuhan, China; 2 School of Electronic and Information Engineering, Beihang University, 100191 Beijing, China

**Keywords:** Integrated optics, Optoelectronic devices and components, Silicon photonics, Magneto-optics, Magneto-optics

## Abstract

Terahertz isolators, one of the typical nonreciprocal devices that can break Lorentz reciprocity, are indispensable building blocks in terahertz systems for their critical functionality of manipulating the terahertz flow. Here, we report an integrated terahertz isolator based on the magneto-optical effect of a nonreciprocal resonator. By optimizing the magneto-optical property and the loss of the resonator, we experimentally observe unidirectional propagation with an ultrahigh isolation ratio reaching up to 52 dB and an insertion loss around 7.5 dB at ~0.47 THz. With a thermal tuning method and periodic resonances, the isolator can operate at different central frequencies in the range of 0.405–0.495 THz. This on-chip terahertz isolator will not only inspire more solutions for integrated terahertz nonreciprocal devices, but also have the feasibility for practical applications such as terahertz sensing and reducing unnecessary reflections in terahertz systems.

## Abstract (web summary)

Nonreciprocal devices are crucial in scientific research and practical applications at all frequencies. Here the authors demonstrate an integrated terahertz optical isolator based on the magneto-optical effect in a nonreciprocal resonator.

## Introduction

Terahertz science and technology have witnessed unprecedentedly rapid development during the past decade and therefore have grown into a disruptive interdisciplinary subject^1–3^, which possesses compelling prospects in fundamental science^3^, wireless communications^4–6^, imaging^7,8^, and sensing^3^. Although recent advances in terahertz frequencies provide both opportunities and challenges, the lack of high-efficiency terahertz sources, high-sensitivity detectors, and functional devices is still the main blocking element hindering the progress of terahertz technology^3,4,9^. Among these components, nonreciprocal terahertz devices hold the possibility of breaking the time-reversal symmetry, consequently realizing the function of irreversible terahertz propagation^10–13^. With nonreciprocity, these devices can constitute terahertz isolators (analogous to diodes) and circulators^13–16^, which has great significance in protecting terahertz sources, mitigating multipath interference, and suppressing undesired signal routing^10,13^.

Recently, researches on terahertz isolators have attracted widespread attention. Traditional terahertz isolators are realized with magnetic-optical materials, which are mostly considered in the following schemes. Schemes based on Faraday effect^14,17^ and magneto-optical Kerr effect^15^ are introduced to design isolators with the help of polarization-converting elements. High-mobility semiconductors (InSb^18–21^, HgTe^22^), graphene^23,24^, ferrofluid^25^, and magnetic materials^14^ are candidates that can be utilized to generate polarization rotation of terahertz waves. The other kind of method is based on the unidirectional absorption^26,27^ and reflection^16,28^, which results from the nonreciprocal directional dichroism^26,27,29–31^ and nonreciprocal reflection loss of the magnetic materials in specific directions^16,28^. However, the schemes above strongly rely on the adjustment of the polarization or the control of the terahertz signal incident angle, therefore, these methods are more suitable for demonstrating terahertz isolators in the process of space transmission. To achieve integrated, compact, and high-efficiency terahertz systems^9,32^, there is an urgent need for the realization of on-chip terahertz isolators, which has not yet been reported.

When we take the performances of reported devices into consideration, terahertz isolators which possess high isolation ratios and small insertion losses are still the main research target^10,13,15,16^. Recently, experiments with great performances for space transmission have been demonstrated to exhibit the isolation function^14–17,26^. In 2016, an isolator based on the magneto-optical Kerr effect of graphene was experimentally demonstrated with an isolation ratio of ~20 dB and an insertion loss of ~7.5 dB, which was suitable for circularly polarized terahertz signals under room temperature and a magnetic field of 7 T^15^. In this work, the large magnetic field limited the practical applications of the device. In 2018, an isolator based on reflection and absorption of InSb was verified with an isolation ratio of 35 dB and an insertion loss of 6.2 dB, which was used for terahertz waves with linear polarization under room temperature and a magnetic field around 0.2 T^16^. For this isolator, further improvements on the isolation ratio would be restricted by the absorption performance of InSb. In this case, terahertz isolators with higher isolation ratios and tolerable insertion losses are still required to be designed and demonstrated, especially for devices working at relatively small external magnetic fields and room temperature.

In this work, we report the design and the demonstration of an on-chip terahertz isolator with ultrahigh isolation ratios for linearly polarized terahertz waves at room temperature. By coupling the magneto-optical material InSb with a terahertz ring resonator, we theoretically and experimentally demonstrate the nonreciprocal transmission. The employment of the nonreciprocal resonator assists in realizing a high isolation ratio with a tolerable insertion loss. To be precise, this component achieves an isolation ratio of 52 dB and the insertion loss around 7.5 dB at ~0.47 THz. Based on an electrically driven thermal tuning method, we tune the isolator beyond a frequency range of a free spectral range (FSR), and witness the nonreciprocal characteristics in the tuning process. In consideration of the periodic resonances of the resonator, we verify the nonreciprocity and the tunability at different central frequencies, which proves that this scheme can be applied to design tunable isolators at required frequencies. Considering the great performance on the isolation and the tunability, the component presented in our work will not only greatly promote the research of terahertz nonreciprocity, but also provide an exhibition of an integrated terahertz isolator for practical applications.

## Results

### Design and simulation

**Fig. 1:**
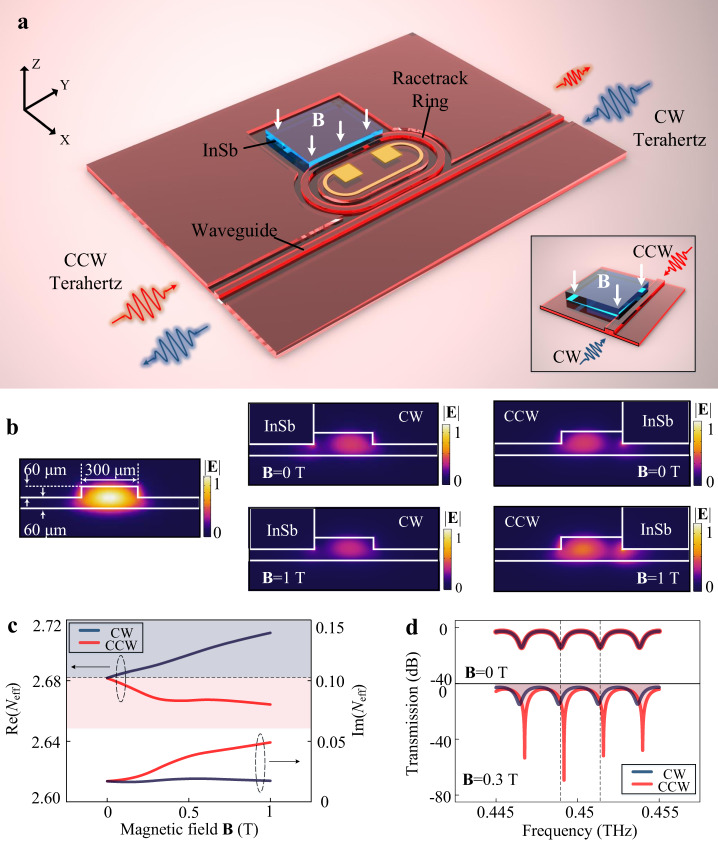
Schematic of the designed on-chip terahertz isolator. **a** Schematic of the on-chip terahertz isolator, including a ridge waveguide, a racetrack ring resonator, a piece of InSb, and a metal electrode (the inset shows the detail of the nonreciprocal region). **b** Simulated normalized electric field distribution (|**E** | ) of the ridge waveguide and the nonreciprocal region (in clockwise (CW) and counterclockwise (CCW) directions). **c** Calculated real (Re(*N*_eff_)) and imaginary (Im(*N*_eff_)) part of effective refractive indices of the nonreciprocal region with the increase of the magnetic field. **d** Transmission properties of the chip in reciprocal (**B** = 0 T) and nonreciprocal states (**B** = 0.3 T). In the calculations, the temperatures are set to 300 K.

Figure 1a illustrates the structure of the proposed terahertz isolator, which includes a straight waveguide, a racetrack ring resonator, a terahertz magnetic material (InSb), and a metal electrode. The racetrack ring resonator and the straight waveguide are both fabricated on a high-resistivity silicon wafer, which possesses ridge waveguide structures in the cross-section to support linear TE polarization^33–36^. Terahertz waves transmit along with the straight waveguide and evanescently excite periodic resonant modes in the ring resonator. To introduce the terahertz magneto-optical effect, we employ a square InSb^13,16,19,37^, which is attached to the outside wall of the straight ridge waveguide in the resonator. On the other hand, the employment of the ring resonator can enhance the magnetic-optical effect of InSb in the chip^38,39^. In this case, the magnetic field will manipulate the phase shift and the loss of the nonreciprocal resonator consisting of the ring and the InSb. The nonreciprocal phase shift leads to different resonant frequencies and the nonreciprocal loss of the resonator will introduce distinct extinction ratios in two directions^40^. For this nonreciprocal resonator, by optimizing the loss and adjusting the state of the resonator, we can obtain tolerable insertion loss and a designable high isolation ratio. Moreover, we fabricate a metal electrode to achieve the function of the electrically driven thermal tuning process ^41,42^.

To verify the property of the proposed nonreciprocal resonator, we simulate the electric field distribution (|**E**|) and the effective refractive indices (*N*_eff_) of the coupling region between InSb and silicon waveguide in both clockwise (CW) and counterclockwise (CCW) directions, as shown in Figs. 1b and 1c. The simulated results indicate that the electric fields in CW and CCW directions transform from symmetric to asymmetric distributions when the magnetic field is applied, and this represents a nonreciprocal behavior. Consequently, with the increase of the magnetic field, the real part of the effective refractive index of CW mode increases from 2.68 to 2.71, while the real part of the CCW mode decreases from 2.68 to about 2.66. As for the imaginary part of the effective refractive index, the CW parameter is almost unchanged, while the CCW direction is increased to about 0.05. Specifically, the model of magnetic material InSb and parameters in the simulations are discussed in Supplementary Note 1.

Nonreciprocal refractive indices lead to direction-related propagation properties, which can be obtained from the calculated transmission spectra shown in Fig. 1d. We employ a transmission model to describe the device, as shown in Eq. (1)^43,44^. In the model, *H* refers to the transmission function of the chip, *a* represents the round-trip transmission coefficient of the ring resonator, *φ* indicates the round-trip phase shift of the resonator, *r* is the self-coupling coefficient between the waveguide and the ring, *m* indicates the direction of the terahertz wave.

1 $${H}_{m}=\frac{{a}_{m}^{2}-2r{a}_{m}\,\cos ({\varphi }_{m})+{r}^{2}}{1-2r{a}_{m}\,\cos ({\varphi }_{m})+{r}^{2}{a}_{m}^{2}},\,(m \sim {{{{{\rm{CW}}}}}},{{{{{\rm{CCW}}}}}})$$

According to the model, the real part of the effective refractive index is related to the round-trip phase shift *φ*, which leads to the change of the resonant frequency. At the same time, the round-trip transmission coefficient *a* changes with the variety of the imaginary part of the effective refractive index, which influences the extinction ratio of the chip. We note that the highest extinction ratio of the resonator occurs at the critical coupling state for a single direction, which represents an equal round-trip transmission coefficient and the self-coupling coefficient (namely *r* = *a*)^44^. Based on calculated results, CW and CCW spectra share the same resonant frequencies and extinction ratios without an applied magnetic field. As the magnetic field is switched to **B** = 0.3 T, the spectrum in the CW direction manifests an obvious decrease of the resonant frequency and an almost unchanged extinction ratio. Meanwhile, in the CCW direction, the resonant frequency increases, and a significant improvement in the extinction ratio from 11.6 dB to near 65.2 dB can be realized. When the operating frequency is fixed at the center of CCW resonance, the transmission loss is 69.3 dB in this direction, and 7.8 dB for the opposite direction, resulting in an isolation ratio of ~61.5 dB. Therefore, we can find that the high extinction ratios and large frequency detuning between resonances in two directions bring an ultrahigh isolation ratio in this chip. More details about the transmission model, designed parameters, and calculated results are carefully considered in Supplementary Note 1.

### Nonreciprocity of the chip

**Fig. 2:**
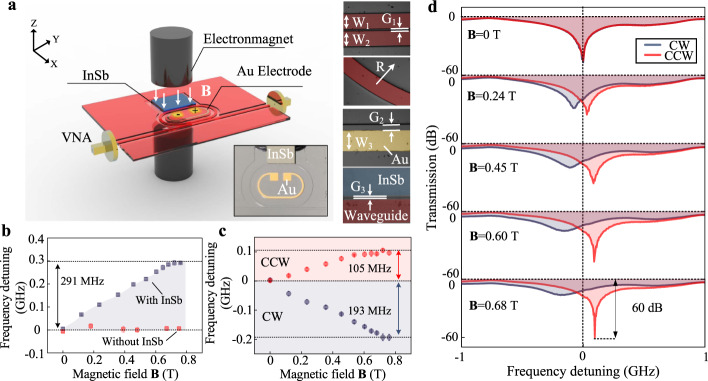
Experimental verification of the terahertz nonreciprocity. **a** The schematic diagram of the experimental setup, and the insets illustrate the photo and optical microscopic images of the chip (*W* refers to the width, *G* refers to the gap, and *R* refers to radius), including the coupling region of two waveguides (marked in red, *W*_1_ = 294.63 µm, *W*_2_ = 292.97 µm, *G*_1_ = 41.68 µm), the bent waveguide (*R* = 4.05 mm), the metal electrode (marked in yellow, *W*_3_ = 314.14 µm, *G*_2_ = 87.31 µm), and the coupling region between the waveguide and the InSb (marked in blue, *G*_3_ = 8.76 µm). **b** Frequency detuning between CW and CCW directions with the change of the magnetic field. Blue points refer to the chip with InSb, while red points refer to the chip without InSb. **c** Frequency detuning of the nonreciprocal chip in CW and CCW directions with the change of the magnetic field. **d** Normalized transmission spectra under different magnetic fields. In **c** and **d**, Blue points and lines refer to CW direction, while red points and lines refer to CCW direction. The error bars in **b** and **c** represent the standard deviations of three measurements of frequency detunings.

Based on the above designs, we fabricated and measured the terahertz isolator, and Fig. 2a illustrates the schematic diagram of the experimental setup. In the measurements, we used an electromagnet to generate a homogeneous magnetic field perpendicular to the device plane, and the poles of the electromagnet were fixed on two sides of the chip^20,21^. Moreover, a magnetometer was used to measure the strength of the field. A vector network analyzer (VNA) with a range of 0.325–0.5 THz was taken to measure the transmission spectra of the chip. During the measuring process, the coupling state of the chip and the VNA were kept invariable, and the transmission direction of the VNA was changed to measure the CW and CCW spectra. More details about the materials, fabrications, and experiments are carefully introduced in Methods and Supplementary Note 2.

We carried out observations on resonant frequencies in CW and CCW directions to record the nonreciprocal properties. First of all, a comparison between devices with and without InSb is taken into consideration, specifically, we use frequency detuning between CW and CCW directions to present measured results, as shown in Fig. 2b. For the device with InSb, with the magnetic field increases from 0 to 0.76 T, frequency detuning significantly increases from smaller than 5 MHz to ~291.67 ± 2.89 MHz. As a comparison, the frequency detuning is smaller than 16.67 ± 11.00 MHz during the experiment for the device without InSb. These results indicate the existence of nonreciprocity when the InSb and the magnetic field are applied. In the measurements, we observed the frequency detuning of CW and CCW transmissions separately with the change of applied magnetic field, as shown in Fig. 2c. For the CW direction, frequency detuning decreases from 3.33 ± 5.78 MHz (**B** = 0 T) to −193.33 ± 11.56 MHz (**B** = 0.76 T), while for the CCW direction, frequency detuning shows an increasing trend from 1.67 ± 2.89 MHz (**B** = 0 T) to 105 ± 5 MHz (**B** = 0.76 T).

Moreover, we also investigated the change of extinction ratios to express the influence on the round-trip transmission coefficient. As shown in Fig. 2d, with the increase of the magnetic field, we illustrate normalized transmission spectra in both CW and CCW directions. On the one hand, the detuning of resonant frequencies can be observed, which is consistent with the results in Fig. 2c. On the other hand, with the magnetic field from 0 to 0.68 T, the extinction ratio of CW resonance decreases from 47 dB to 16.3 dB, while CCW increases from 47 dB to 60 dB. Considering that the largest extinction ratio exists at the critical coupling state of a resonator, we can safely draw a conclusion that, for the CW direction, the coupling state gradually deviates from the critical coupling state, while the state of the CCW direction moves towards the critical coupling. We note that, in the practical experiment, there is a gap between the InSb and silicon waveguide, which influences properties of the nonreciprocal region, and we theoretically discuss the property of the chip with a gap in Supplementary Note 3.

### Terahertz isolations

**Fig. 3:**
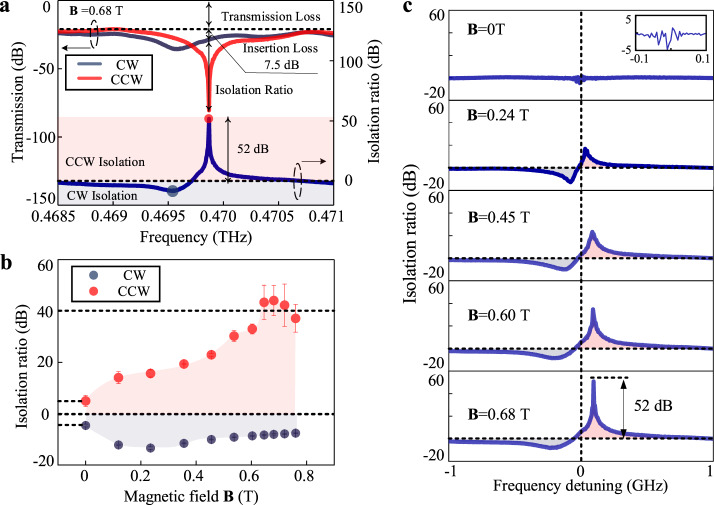
Performance characterization of the terahertz isolator. **a** Transmission spectra of the chip when **B** = 0.68 T, and the isolation ratios are defined as *H*_CW_–*H*_CCW_; the positive isolation ratio indicates that the loss in CW direction is smaller while the negative isolation ratio refers to the larger loss in CW direction. **b** Measured maximum isolation ratios of positive and negative states with the change of the magnetic field. The error bars represent the standard deviations of three measurements of isolation ratios. **c** Isolation spectra of the chip in the change of the magnetic field.

Based on the successful observation of nonreciprocal transmission, we investigate the performances of the device acting as an isolator. Figure 3a illustrates the transmission spectra and calculated terahertz isolation ratio at ~0.47 THz. The transmission loss in non-resonant frequencies, which mainly comes from the coupling loss of the chip and partly from the propagating loss of the waveguide and the resonator with InSb, is ~21 dB in total. To consider the isolator performance, we set the operating frequency at the central resonant frequency of the CCW direction^40^. In this case, an isolation ratio of 52 dB is obtained, with an insertion loss of around 7.5 dB. We note that the loss of ~7.5 dB does not include the coupling loss and the transmission loss (related to waveguide and InSb)^40^. Incorporation with measurements and simulations, the insertion loss should be between 7.5 and 12.2 dB when the transmission loss related to InSb is considered (See Supplementary Note 3 for detailed discussions). Then we calculate the isolation ratio (Isolation Ratio = *H*_CW_ − *H*_CCW_) to figure out its performance at different central frequencies. The negative results indicate CW isolations, marked in the blue region, which refers that the propagating loss in the CW direction is larger than the CCW direction. While the positive results, marked in red, indicate CCW isolations, which represent a larger propagating loss in the CCW direction. Since the extreme isolation ratios occur at resonant frequencies of CW and CCW directions, we have a CW isolation of 8.21 dB at 0.46954 THz and a CCW isolation of 52 dB at 0.46987 THz. Moreover, the isolator bandwidth is limited by the bandwidth of the ring resonator and the frequency detuning, computationally, we obtain a frequency range of 0.14 GHz, which has an isolation ratio of more than 10 dB.

Figure 3b shows maximum isolation ratios in CW and CCW directions with the variation of the magnetic field at ~0.47 THz. It should be mentioned that the isolation between the two directions should be completely 0 dB when **B** = 0 T. However, the experimental results of the isolation ratios (<±5 dB) can be attributed to the measuring deviation. We note that CW and CCW spectra are not measured at the same time strictly, which introduces a slight deviation between the central frequency and the extinction ratio, and therefore nonzero values are attained. With an increased magnetic field larger than 0.1 T, the CCW isolation is larger than 15 dB, and the average isolation ratios exceed 40 dB when **B** > 0.6 T. Moreover, the measured maximum CCW isolation ratio is 52 dB when **B** = 0.68 T. For the CW direction, the isolation ratios first gradually increase and then decrease, which are below 20 dB in the whole measurements.

Next, we illustrate the isolation ratio spectra at different applied magnetic fields, as shown in Fig. 3c. When **B** = 0 T, the calculated result of the device is below ±5 dB, and the spectra illustrate irregular oscillations rather than a continuous variation, indicating that the deviation is due to the measurement jitter. When **B** > 0 T, the isolation ratio changes continuously with the increase of the frequency, indicating the existence of the isolation function. As the maximum isolation frequency mainly appears at the resonant frequencies, we can find that the extreme values of the spectra change and move away from zero detuning, which agrees with the results in Fig. 3b.

### Tunability of the isolator

**Fig. 4:**
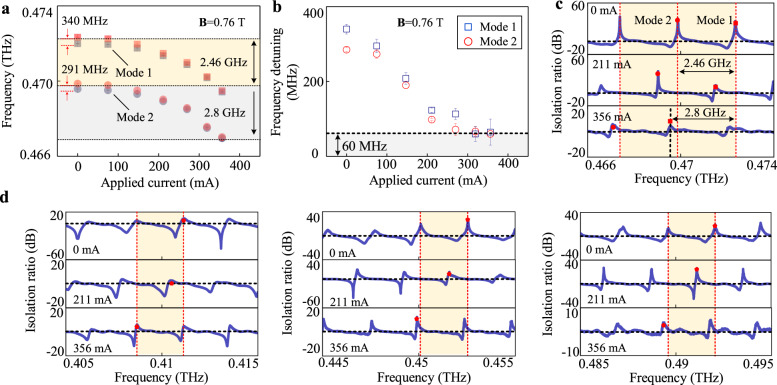
Electrically driven tunability of the terahertz isolator. **a** Resonant frequencies of two adjacent modes with the change of the applied current when **B** = 0.76 T. The error bars represent the standard deviations of three measurements of central frequencies. In **a**, blue dots refer to the CW direction and red dots refer to the CCW direction. In **a** and **b**, the square points are marked as mode 1, and the circle points refer to mode 2. **b** Frequency detuning between CW and CCW directions (*F*_CCW_–*F*_CW_: *F* refers to the resonant frequencies) with the variety of the applied current when **B** = 0.76 T. The error bars represent the standard deviations of three measurements of frequency detunings. **c** Isolation ratios of the chip in the change of the applied current (0, 211, 356 mA). **d** Isolation ratios in the change of the applied current (0, 211, 356 mA) at different operation frequencies, frequency windows are 0.405–0.415, 0.445–0.455, and 0.485–0.495 THz.

As mentioned before, the bandwidth of this isolator is limited by the bandwidth of the ring structure, therefore, this isolator should be tunable to fulfill the requirements of real-world applications. In the experiment, we modulated the central frequency of the isolator via employing an electrically driven thermal tuning method^35,41^, enabling the chip adjustability. Meanwhile, the magnetic field is utilized to introduce the nonreciprocal property of the chip. Figure 4a illustrates the tunability of the nonreciprocal resonator. When **B** = 0.76 T, the nonreciprocal resonator can be obtained and verified since the CW and CCW resonances have distinct central frequencies. Since the resonances are periodic for the resonator, the round and square points represent two adjacent resonant modes, and the FSR is ~2.46 GHz. As the applied current increases, the local temperature of the device rises, and the effective refractive index of the corresponding mode increases, therefore the resonant frequency of the device gradually decreases. With the applied current exceeds 365 mA, the tuning range of the resonance reaches up to ~2.8 GHz, which is larger than a single FSR. Meanwhile, the frequency detuning decreases when the applied current increases with the minimum detuning of ~60 MHz in the tuning process, as shown in Fig. 4b, which verifies that the isolation function still exists in the tuning process. The function is further observed by calculating the isolation ratios at different currents, shown in Fig. 4c, and the central frequency of the isolator can be tuned beyond a frequency range of a single FSR with different maximum isolation ratios.

Taking the periodic resonances into account, the isolation ratio can be observed in different frequency regions. Figure 4d illustrates the isolation ratios in three frequency ranges of 0.405–0.415, 0.445–0.455, and 0.485–0.495 THz. For the first, resonances and nonreciprocity can be observed in all frequency ranges. Second, tuning of nonreciprocal transmission can also be realized and the tuning ranges are larger than a single FSR. As a result, this isolator can operate within a range of 0.405–0.495 THz. When we optimize the parameters of the chip, this tunable isolator can be used for specific frequencies between 0.405 and 0.495 THz in practical applications (See Supplementary Note 4 for detailed discussions).

## Discussion

We compare the performances of reported works and this work, our isolator has advantages in properties of integration and isolation ratio, as shown in Table 1. Meanwhile, this isolator possesses the property of flexible tunability on the operating frequency. In considerations of the parameters listed above, the isolator can be utilized in situations which can tolerate small bandwidths. In real-world applications, this chip can be applied in protecting tunable continuous-wave terahertz sources with narrow linewidths^10,45^, introducing functions as a filter or a temporal differentiator for processing terahertz signals with a modulating speed of megahertz level^35,36^, realizing applications for terahertz sensing such as detection of magnetic fields^46^.

**Table. 1:** Comparison between reported terahertz isolators and the isolator in this work.

Time	Integration	Maximum isolation ratio (dB)	Typical insertion loss (dB)	Temperature	Magnetic field (T)	Bandwidth	Tunability of central frequencies
2013^14^	–	–	–	Room	0.54	Wideband	–
2016^15^	–	~20	~7.5	Room	7	50 GHz	–
2018^16^	–	35	6.2	Room	0.2	45 GHz	–
2019^18^	–	20	~10	200 K	0.15	>0.5 THz	–
2020^17^	–	18.8	12.6	Room	0.14	–	–
This work	√	52	~7.5	Room	0.68	0.14 GHz	√

In conclusion, we propose and experimentally demonstrate an on-chip terahertz isolator based on a nonreciprocal resonator, which includes a silicon ring resonator and InSb. The property of this nonreciprocal resonator is systematically studied, and the frequency detuning between CW and CCW transmissions is observed. The isolation function is successfully realized with the applied magnetic field from 0 to 0.8 T, which has a 52 dB ultrahigh isolation ratio and an insertion loss around 7.5 dB for the best. Furthermore, the isolator can be tuned by an electrically driven thermal tuning method, and a tuning range of more than 2.46 GHz (FSR) can be realized. Based on the periodic resonances, this isolator can be utilized to work in the frequency range of 0.405–0.495 THz. As fundamental devices in terahertz systems, isolators proposed in this work will contribute to the realization of other kinds of terahertz nonreciprocal devices such as circulators, and have great potential for applications, such as terahertz sensing, nonreciprocal terahertz routing, and source protection, in different kinds of terahertz systems.

## Methods

### Materials

A 2 inch high-resistivity silicon wafer (resistivity greater than 10 kΩ•cm) with a thickness of 120 μm is utilized to fabricate the ridge waveguide and ring resonator, which has a relatively high refractive index around 3.42 and a low absorption coefficient smaller than <0.025 cm^−1^ when the frequency is lower than 1 THz^33,35^. As for InSb, we use a 2-inch wafer with a thickness of 500 μm to fabricate the device. This wafer is a Te N-type doped wafer, which has a carrier concentration of 5 × 10^14^–9 × 10^16^ cm^−3^ and a crystal orientation of <100> direction.

### Fabrications

The fabrication process of the device could be divided into the following steps^35^. First of all, with the help of ultraviolet (UV) lithography (MJB4) and electron beam evaporation (Ebeam500S), the gold electrode and gold alignment marks are prepared for the next UV lithography. In this step, to improve the adhesion between gold and high-resistivity silicon, a Ti layer, which has a 20 nm thickness is deposited first and then a deposition step of 140 nm gold is accomplished. After that, the second step of UV lithography and inductively coupled plasma etching (ICP, Oxford Plasmalab System 100 ICP108) are used to fabricate the silicon waveguide and the ring resonator.

The InSb wafer is cleaved to an InSb square with a side length of 5 mm and a thickness of 500 μm. On the fabricated high-resistivity silicon chip, the InSb is attached outside the straight waveguide of the resonator by a bonding method, and the gap is controlled as close as possible, which is 8.76 μm in the above measurements. Please see Supplementary Note 2 for more details.

### Measurements

In the experiment, a vector network analyzer (VNA, Ceyear 3649B) with a frequency range of 0.325–0.5 THz is utilized to measure the device. The input and output ports of the VNA can be switched automatically, and therefore we measure CW and CCW spectra by adjusting the direction of the terahertz wave without changing the coupling state of the chip. The terahertz signal is coupled to the waveguide in the TE polarization through the output metal waveguide of the network analyzer (internal size 508 × 254 μm^2^, meeting WR-2.2 standard), and then the terahertz signal is coupled back to the metal waveguide of the VNA from the silicon waveguide on the other side.

During the test, the magnetic field is introduced by an electromagnet whose magnetic poles are two cylinders with diameters of 20 mm, and two poles are placed at the upper and lower sides of the InSb area of the chip. By adjusting the gap of two magnetic pole heads, an appropriate distance is set to introduce a proper magnetic field. The electromagnet is controlled by a voltage source, which can be precisely adjusted, and a magnetometer is used to measure the magnetic field at a specific voltage. To tune the device, a tunable current is introduced to the device through a voltage source to generate the electric thermal effect.

## Supplementary information

Supplemetary Information for On-chip terahertz isolator with ultrahigh isolation ratios

## Data Availability

The authors declare that all the data supporting this study are available within the manuscript and the Supplementary Information. Additional data related to this article are available from the corresponding authors upon reasonable request.

## Code availability

Any simulation and computational codes for this study are available from the corresponding authors on reasonable request.
