# Supplementary material for: On-chip terahertz isolator with ultrahigh isolation ratios
Source: Nat Commun. 2021 Sep 22;12:5570. doi: 10.1038/s41467-021-25881-0 (PMC8458294; doi:10.1038/s41467-021-25881-0)
Supplement: Supplementary file 1 — Supplemetary Information for On-chip terahertz isolator with ultrahigh isolation ratios [file 41467_2021_25881_MOESM1_ESM.pdf]

Supplementary Information for

On-chip terahertz isolator with ultrahigh isolation ratios

Shixing Yuan,[1] Liao Chen,[1] Ziwei Wang,[1] Wentao Deng,[1] Zhibo Hou,[1] Chi Zhang,[1] Yu Yu,[1] Xiaojun Wu[2,1,*], and Xinliang Zhang[1,**]

[1]Wuhan National Laboratory for Optoelectronics and School of Optical and Electronic Information, Huazhong University of Science and Technology, Wuhan 430074, China

[2]School of Electronic and Information Engineering, Beihang University, Beijing 100191, China

Corresponding author: [*]xiaojunwu@buaa.edu.cn, [**]xlzhang@mail.hust.edu.cn

Supplementary Note 1: Theoretical model and calculations

S1.1 Theoretical model of InSb

We employ InSb, which is a semiconductor with high electron mobility, to generate magneto-optical nonreciprocity. As a typical magnetic material at the terahertz frequency, the material possesses an asymmetric dielectric constant matrix, in which these components strongly depend on the temperature and external magnetic field. Specifically, the anti-angle elements are proportional to the magnetic field. The dielectric permittivity of InSb can be described by the following Supplementary Equations (1)~(5), where the magnetic field is along the z-direction.[1-4]

$$\varepsilon=\begin{bmatrix}\varepsilon_{\mathrm{xx}} & \varepsilon_{\mathrm{xy}} & 0\\ \varepsilon_{\mathrm{yx}} & \varepsilon_{\mathrm{yy}} & 0\\ 0 & 0 & \varepsilon_{\mathrm{zz}}\end{bmatrix} \tag{1}$$

$$\varepsilon_{\mathrm{xx}}=\varepsilon_{\mathrm{yy}}=\varepsilon_{\infty}-\frac{\omega_{\mathrm{p}}^{2}\left(\omega+\mathrm{i}\gamma\right)}{\omega\left[\left(\omega+\mathrm{i}\gamma\right)^{2}-\omega_{\mathrm{c}}^{2}\right]}+\varepsilon_{\mathrm{ph}} \tag{2}$$

$$\varepsilon_{\mathrm{xy}}=-\varepsilon_{\mathrm{yx}}=\frac{\mathrm{i}\omega_{\mathrm{p}}^{2}\omega_{\mathrm{c}}}{\omega\left[\left(\omega+\mathrm{i}\gamma\right)^{2}-\omega_{\mathrm{c}}^{2}\right]} \tag{3}$$

$$\varepsilon_{\mathrm{zz}}=\varepsilon_{\infty}-\frac{\omega_{\mathrm{p}}^{2}}{\omega^{2}+\mathrm{i}\omega\gamma}+\varepsilon_{\mathrm{ph}} \tag{4}$$

$$\varepsilon_{\mathrm{ph}}=\varepsilon_{\infty}\left(\frac{\omega_{\mathrm{t}}^{2}-\omega_{\mathrm{l}}^{2}}{\omega_{\mathrm{t}}^{2}-\omega^{2}-\mathrm{i}\gamma_{\mathrm{ph}}\omega}\right) \tag{5}$$

$$n\left(\mathrm{cm}^{-3}\right)=n_{0}+2.9\times10^{11}\left(2400-T\right)^{\frac{3}{4}}\left(1+2.7\times10^{-4}T\right)T^{\frac{3}{2}}\times\exp\left(\frac{-\left(0.129-1.5\times10^{-4}T\right)}{k_{\mathrm{b}}T}\right) \tag{6}$$

In these equations, $\omega_{\mathrm{p}}=ne^{2}/(m^{*}\varepsilon_{0})$ represents the plasma frequency, which depends on the carrier concentration n and the equivalent mass m^{*}. We note that the n could be expressed by Supplementary Equation (6), which corresponds to temperature T. k_{b} is

the Boltzmann constant and n_0 is the intrinsic doping carrier density.[2] γ refers to the carrier scattering rate, and ε_∞=15.6 is the background high-frequency dielectric constant. ω_c=$e\mathbf{B}/m^*$ denotes the electron cyclotron frequency determined by the magnetic field. The contribution of phonons to the function can be expressed in the Supplementary Equation (5). ω_t and ω_l describe horizontal and vertical optical phonon frequency, respectively, and γ_{ph} represents the phonon damping rate.[1,2]

According to the model, when the applied magnetic field is **B**=0 T, we have ω_c=0, and therefore ε_{xy}=-ε_{yx}=0. In this case, the matrix in Supplementary Equation (1) becomes a symmetric matrix, which indicates that the InSb is a reciprocal material. When the applied magnetic field **B**>0 T is considered, ε_{xy} and ε_{yx} are not 0. We have an asymmetric dielectric constant matrix, and therefore the nonreciprocity is introduced.

S1.2 Transmission model of the chip

To characterize the transmission property of the device, a transmission model based on the transfer matrix method is adopted, as shown in Supplementary Fig. 1a. The transfer matrix of electric fields at different locations is described by the following Supplementary Equations (7)~(8). In these equations, r indicates the self-coupling coefficient between the waveguide and the ring. Correspondingly, the coupling coefficient κ can be expressed as κ^2=1-r^2. Parameter L represents the length of the straight waveguide in the resonator, and R is the radius of the bending waveguide. As to the ring resonator, a denotes the round-trip transmission coefficient, and φ describes the round-trip phase shift. Solving the Supplementary Equations (7) and (8), the transfer

function of a certain direction can be calculated by H=|h|^2,[5,6] as shown in the Supplementary Equation (9) and the Equation (1) in the main text. It should be noted that the parameters such as a and φ are composed of parameters in several parts, including the straight waveguide, the bending waveguide, and the nonreciprocal area. In this case, we utilize Supplementary Equations (10) and (11) to calculate these parameters, in which N_{eff1}, N_{eff2}, and N_{eff} refer to the effective refractive index of the ridge waveguide, the curved waveguide, and nonreciprocal region, respectively. m refers to the direction of the terahertz wave, including clockwise (CW) and counterclockwise (CCW) directions. To obtain a and φ theoretically, the finite element method should be used to calculate the effective refractive index at different positions. Moreover, $\mathbf{k}_0$ is the propagation constant under vacuum.

$$\begin{bmatrix} \mathbf{E}_1 \\ \mathbf{E}_{\mathrm{out}} \end{bmatrix} = \begin{bmatrix} r & -\mathrm{i}\kappa \\ -\mathrm{i}\kappa & r \end{bmatrix} \begin{bmatrix} \mathbf{E}_2 \\ \mathbf{E}_{\mathrm{in}} \end{bmatrix} \tag{7}$$

$$\mathbf{E}_2 = \mathbf{E}_1 \bullet \left(a \cdot \exp(\mathrm{i}\varphi) \right) \tag{8}$$

$$h = \frac{\mathbf{E}_{\mathrm{out}}}{\mathbf{E}_{\mathrm{in}}} = \frac{r - a \cdot \exp(\mathrm{i}\varphi)}{1 - ra \cdot \exp(\mathrm{i}\varphi)} \tag{9}$$

$$\varphi_m = \mathbf{k}_0 \cdot \left(L \cdot \mathrm{Re}\left(N_{\mathrm{eff1}}\right) + 2\pi R \cdot \mathrm{Re}\left(N_{\mathrm{eff2}}\right) + L \cdot \mathrm{Re}\left(N_{\mathrm{eff},m}\right) \right) \tag{10}$$

$$\mathrm{In}\left(a_m\right) = -\mathbf{k}_0 \cdot \left(L \cdot \mathrm{Im}\left(N_{\mathrm{eff1}}\right) + 2\pi R \cdot \mathrm{Im}\left(N_{\mathrm{eff2}}\right) + L \cdot \mathrm{Im}\left(N_{\mathrm{eff},m}\right) \right) \tag{11}$$

In the calculation, the mode conversion loss in the resonator, coupling loss between the chip and the waveguide of Vector Network Analyzer (VNA), and the transmission loss of the straight waveguide are not considered in this model. The real part of the effective refractive index is related to the round-trip phase shift of the ring. When $\varphi=l\bullet 2\pi$ (l=1,2,3……) is achieved, resonance occurs and the resonant frequency can be

obtained. With the increase of N_{eff}, the resonant frequency of the resonator will decrease. The imaginary part of the effective refractive index will change the round-trip transmission coefficient a. Considering that the parameter r is a constant in a single structure, with different a, the chip will have different extinction ratios. It should be noted that the extinction ratio depends on the relationship between r and a. When r=a, the chip is in the critical coupling state. Consequently, it possesses the highest extinction ratio.

S1.3 Additional details and calculated results

In the calculations, we use the finite element method to simulate the effective refractive indices. Firstly, a waveguide is designed with a width of 300 μm, a ridge height and a substrate height of 60 μm, as shown in Fig. 1b, which can support linear TE mode. As to InSb, we utilize a 500 μm-thick wafer in the experiment, therefore the same parameter is considered in the simulations. Moreover, the temperature is set to T=300 K in all simulations. To obtain a better performance, we selected L=4 mm, R=4 mm, and a waveguide gap of around 35 μm as the resonator parameters.

According to the transmission model, we can calculate the transmission spectra of the device. When r=a, the resonator is in a critical coupling state, and the mismatch between r and a will leads to an obvious decrease of the extinction ratio. As shown in Fig. 1d in the main text, we have the parameter of r=0.35. In the calculation when $\mathbf{B}$=0 T, the round-trip transmission coefficient is calculated to be a=0.508 in both CW and CCW directions. Therefore, two spectra have the same resonant frequencies and

extinction ratios in the over coupling state (namely $a>r$). When **B**=0.3 T, we have a_{CW}=0.4988 and a_{CCW}=0.3537. In this case, CCW resonance is nearly a critical coupling state which possesses a high extinction ratio up to 65.2 dB, while the CW resonance is still in an over coupling state. With the change of the parameter r, the state of the resonator can be designed, as shown in Supplementary Fig. 1b. When r is set to r=0.4988, CW resonance is changed to a nearly critical coupling state. In this case, with the applied magnetic field **B**=0.3 T, a CW resonance with a high extinction ratio up to 58.7 dB can be obtained, and CCW resonance possesses an extinction ratio of around 12.2 dB in an under coupling state (namely $a<r$).

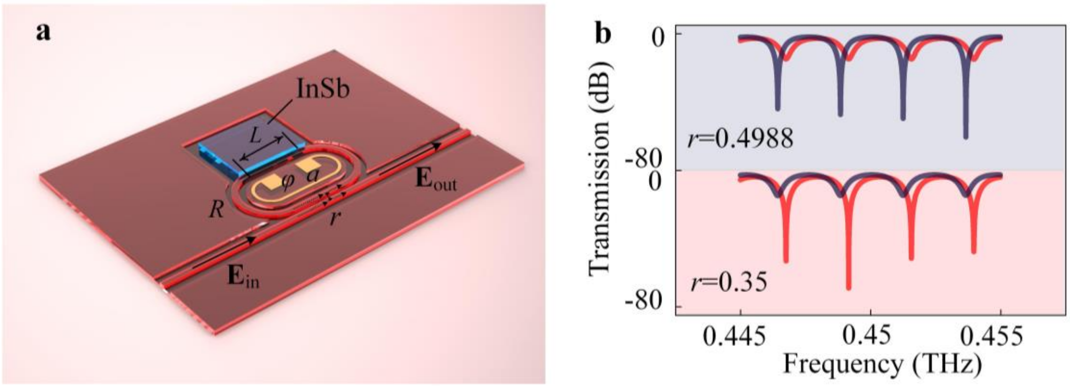

Supplementary Figure 1. Transmission module of the chip and supplementary calculation results. a The transmission model of the chip. **b** Calculated results when r=0.4988, 0.35, **B**=0.3 T. r=0.4988 refers to a high extinction ratio in the CW direction (marked in blue region), while r=0.35 represents a high extinction ratio in the CCW direction (marked in red region). Blue lines refer to CW direction, while red lines refer to CCW direction.

Therefore, it is reasonable to have a high extinction ratio parameter in one of the CW and CCW directions, which only depends on the match of the round-trip transmission coefficient a of the specific direction and self-coupling coefficient r of the waveguide coupling region. Based on the high extinction ratio occurring in a specific direction, ultrahigh isolation ratios can be realized.

Supplementary Note 2: Fabrication and measurement of the chip

S2.1 Fabrication of InSb

In the method part, the main fabrication progress of the high-resistivity silicon chip is discussed, and here is a brief description of the bonding process of InSb, as shown in Supplementary Fig. 2. We select a bonding method to fix the position of the InSb. In the process, a two-inch InSb wafer with a thickness of 500 μm is firstly cleaved to obtain a square piece with a size of 5 mm×5 mm. Next, the square InSb locates at the outside of the ring resonator, and the position is adjusted close to the sidewall of the waveguide. Finally, the InSb can be fixed by using ultraviolet glue.

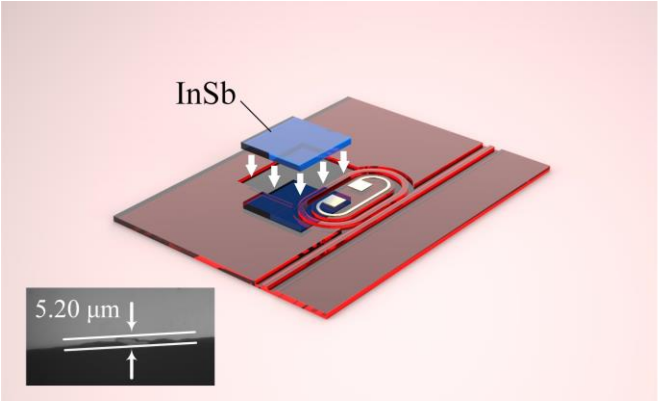

Supplementary Figure 2. Schematic diagram of localizing the InSb on the silicon chip. The lower left illustration is a photomicrograph of the edge of the InSb piece after cleavage.

It should be noted that the InSb has a smooth edge after cleaving, as shown in the inset, the largest fluctuation is 5.2 μm. In the experiment, we observe the smallest gap between InSb and the silicon waveguide to discuss the state of the device.

S2.2 Experimental setup

To clearly illustrate the experimental setup, we show photos of the system in the measurements, see Supplementary Fig. 3. In the experiment, the adjustment of the electromagnet is realized by an accurate voltage source. Three-dimensional high-precision translation stages are utilized to achieve precise positioning and device coupling of the VNA modules and the chip. Moreover, the applied current of the chip is also controlled by the voltage source to tune the central frequency of the resonance.

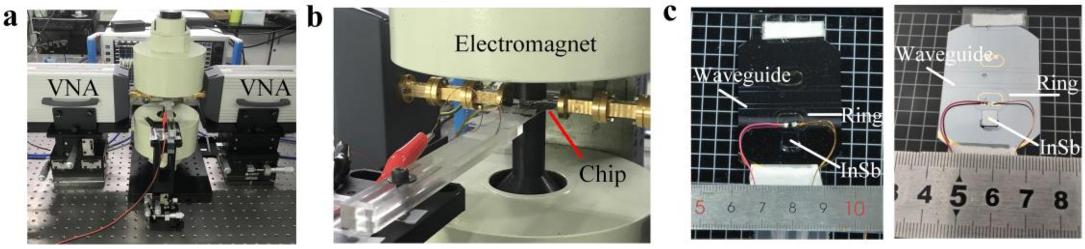

Supplementary Figure 3. Photos of experimental system and device. a Diagram of the experimental setup. **b** Photo of device area. **c** Photos of the terahertz isolator chip.

Supplementary Note 3: Additional discussions of chips with InSb

In the experiment, there is a gap between InSb and the side of the ridge waveguide. The gap will decide the actual behavior of the chip, which is slightly different from the ideal case in Fig. 1 and Supplementary Note 1. Here, additional simulations and measurements are carried out to discuss the property of the chip with a gap.

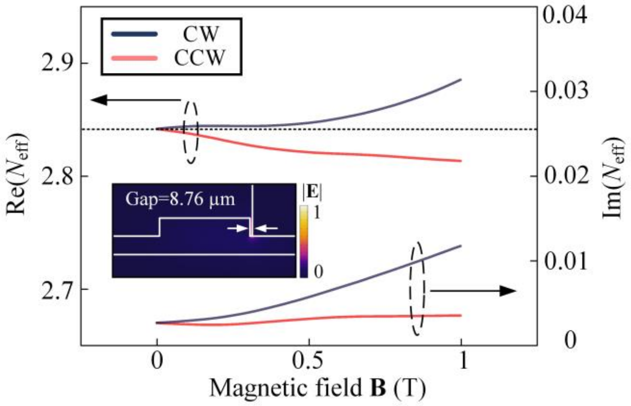

Supplementary Figure 4. Calculated real (Re(N_{eff})) and imaginary (Im(N_{eff})) part of effective refractive indices of the nonreciprocal region with the increase of the magnetic field when the gap is ~8.76 μm. The inset shows the normalized electric field (|**E**|) distribution of the nonreciprocal region.

First of all, the performances of the chip in the main text are discussed based on both theory and measurements. In the bonding process, the gap between InSb and silicon waveguide could not be controlled to be zero, especially when we consider fluctuations at the edge of InSb. We measured the minimum distance between the InSb and the waveguide, which is ~8.76 μm, as shown in Fig. 2a in the main text. We

simulated the refractive indices of the nonreciprocal region, shown in Supplementary Fig. 4. The real part of the effective refractive index in the CW direction increases as the magnetic field increases, while the index decreases in the CCW direction. Correspondingly, the resonant frequency of the CW direction decreases and the CCW frequency increases. At the same time, the mode loss in the CW direction significantly increases while it has a smaller change in the CCW direction, which is different from the ideal model shown in Fig. 1 in the main text. Therefore, the extinction ratio of the resonance varies drastically in the CW direction, as shown in Fig. 2d. Based on the transmission model, simulated spectra further validate the above analysis, as shown in Supplementary Fig. 5a and 5b. When **B**=0 T, the extinction ratios of CW and CCW resonances are around 24.4 dB. While the CW and CCW extinction ratios are 5.2 dB and 57 dB respectively when **B**=0.68 T. The decrease of CW extinction ratios and increase of CCW extinction ratios are consistent with the changes in the experiment shown in Fig. 2.

Meanwhile, the introduction of InSb will influence the transmission loss of the chip. In Supplementary Fig. 5b and 5c, transmission losses are 2.5 dB for CW direction and 1.0 dB for CCW direction, which mainly comes from the influence of InSb. For the isolator, the transmission loss related to InSb should be discussed when we consider the insertion loss, which refers to the total loss in the CW direction (6.6 dB) when the operating frequency is set to the center of the CCW resonance. And the total loss can be divided into two parts, shown in Supplementary Fig. 5c: (1) the part which equals to CCW transmission loss (1 dB); (2) the part which contains CW resonant loss and part

of CW transmission loss (5.6 dB). Therefore, we can find that the latter takes up the major part of the insertion loss. Similarly, in the experiment, ~7.5 dB corresponds to part (2), which takes up the major part of the insertion loss, and the part which equals CCW transmission loss is not included.

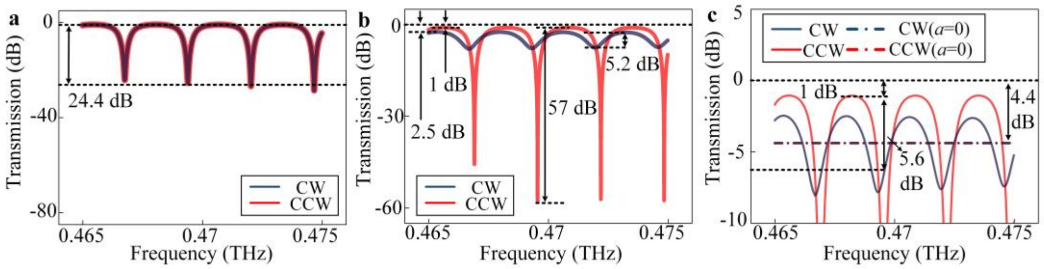

Supplementary Figure 5. Simulated spectra with a gap (~8.76 μm) between the ring and InSb. a Calculated spectra when **B**=0 T. **b** Calculated spectra when **B**=0.68 T. **c** Detailed spectra of the chip with InSb when **B**=0.68 T. Parameters: T=300 K, solid lines in **a**~**c** r=0.602, and dash-dot lines in **c** are r=0.602, a=0. In the calculation, the length of the nonreciprocal region is set to 1.48 mm to be closer to reality based on the frequency detuning.

Moreover, we discuss a special case to estimate the range of the insertion loss with CCW transmission loss. In theory, when all terahertz signals lose in the ring (the round-trip transmission coefficient (a) is zero), the transmission loss shown in Supplementary Fig. 5c is 4.4 dB, which is significantly larger than the CW and CCW transmission loss. In the experiments, it is difficult to distinguish CCW transmission loss from other losses such as coupling loss. Therefore, we provide a range of insertion loss incorporation for both experiments and simulations. Based on the parameters of the chip (shown in Fig.

2) and Lumerical FDTD solutions, the parameter *r* of the device is around 0.583 at 0.47 THz. When *a*=0 we obtain a loss of 4.7 dB, which should be significantly larger than CCW transmission loss. In this case, we can safely draw a conclusion that the insertion loss of ~7.5 dB (in which CCW transmission loss is not included) takes up the major part of the insertion loss, and the insertion loss should be between 7.5 dB and 7.5+4.7=12.2 dB when we consider CCW transmission loss.

Secondly, additional simulations and measurements are carried out to discuss the influence of InSb in resonant frequencies with varied gaps. The relative changes of effective refractive indices (N_{eff} ($\mathbf{B}$=0.78 T) – N_{eff} ($\mathbf{B}$=0 T)) in CW and CCW directions with the increase of gap are simulated, as shown in Supplementary Fig. 6a. When the gap is relatively small (<12.5 μm), effective refractive indices increase in CW direction and decrease in CCW direction. While the indices of CW and CCW directions both decrease as gaps are relatively large (>12.5 μm). These indicate that the existence of InSb can lead to different frequency detuning when there is a gap. As to the distribution of electric fields, when the gap is less than 2.5 μm, the mode profile is mainly distributed in the silicon waveguide, as illustrated in Supplementary Fig. 6b. And the mode field is mainly distributed in the gap at the corner of InSb when the gap is larger than 5 μm. When the gap is further improved, the distribution in the waveguide gradually increases as the InSb has weak coupling strength with the waveguide. We experimentally measured two more chips and the results are illustrated in Supplementary Fig. 6c (gap=7.7 μm) and 6d (gap=15.67 μm). When the gap is 7.7 μm, the resonant frequencies in CW and CCW directions shift in two directions and the

extinction ratio of CW direction possesses a larger decrease, which is similar to measurements in the main text. When the gap is 15 μm, the resonances in both CW and CCW directions move towards the higher frequencies, which is consistent with the simulation in Supplementary Fig. 6a.

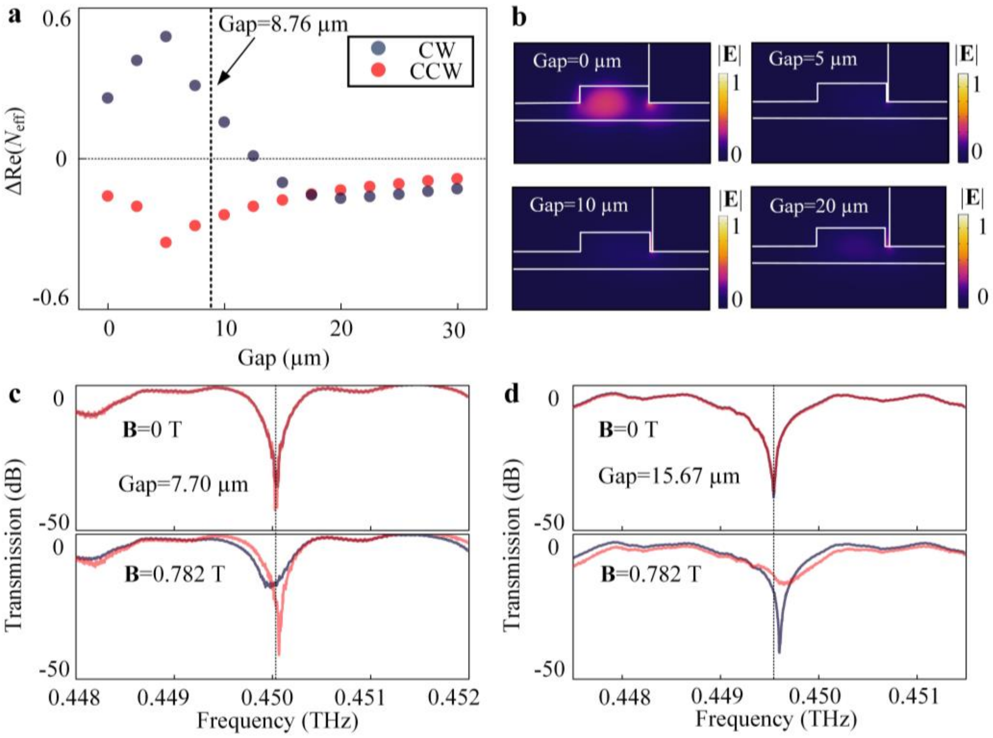

Supplementary Figure 6. a Calculated differences of the real parts of effective refractive indices ($\Delta\mathrm{Re}(N_{\mathrm{eff}})=N_{\mathrm{eff}}$ (**B**=0.78 T) – N_{eff} (**B**=0 T)) with the change of gap between InSb and silicon waveguide in both CW and CCW directions. **b** Simulated normalized electric field (|**E**|) distribution of the nonreciprocal region when **B**=0 T in the CCW direction with the change of the gap. **c**~**d** Measured transmission properties in reciprocal (**B**=0 T) and nonreciprocal (**B**=0.782 T) states, and the gaps of **c** and **d** are 7.7 μm and 15.67 μm. Blue points and lines refer to CW direction, while red points and

lines refer to CCW direction.

Supplementary Note 4: Tunability of the chip

S4.1 Thermal tuning process of the chip

In Fig. 4a of the main text, we observe the tuning of nonreciprocal state when **B**=0.76 T. While the chip is in a reciprocal state for the case of **B**=0 T, and the tuning process is also observed in the experiment, as shown in Supplementary Fig. 7. In this case, CW and CCW resonances share the same resonant frequencies for two observed modes, which verifies the reciprocal state of the chip. In the tuning process, the resonant frequencies decrease with the increase of applied current. The total tuning range is ~2.8 GHz, which is beyond the frequency range of a single FSR ~2.46 GHz.

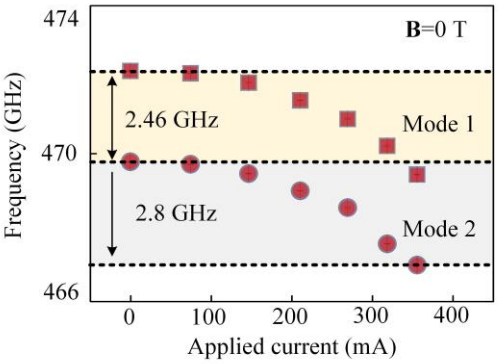

Supplementary Figure S7. Resonant frequencies of two adjacent modes with the change of the applied current when **B**=0 T. The square points are marked as mode 1, and the circle points refer to mode 2; blue dots refer to the CW direction and red dots refer to the CCW direction. The error bars represent the standard deviations of three measurements of resonant frequencies.

Meanwhile, the chip temperature varied with the introduction of the applied current. During this process, the frequency detuning mainly comes from the thermal-optical effect of the silicon ring. In this case, the temperature-dependent frequency detuning can be described by the Supplementary Equation (12).[6,7] With measured frequency detuning, we can obtain the temperature around the ring structure.

$$\Delta F = -\Delta T \cdot \frac{F_0}{N_c} \cdot \left(\frac{\Delta N}{\Delta T} \right) \tag{12}$$

In the equation, ΔF refers to the frequency detuning, and ΔT represents the variation of the temperature. F_0 denotes the central frequency of the resonance, and N_c is the group refractive index. Meanwhile, $\Delta N/\Delta T$ refers to the thermal-optical coefficient of the material. In this calculation, we mainly consider the material of silicon.

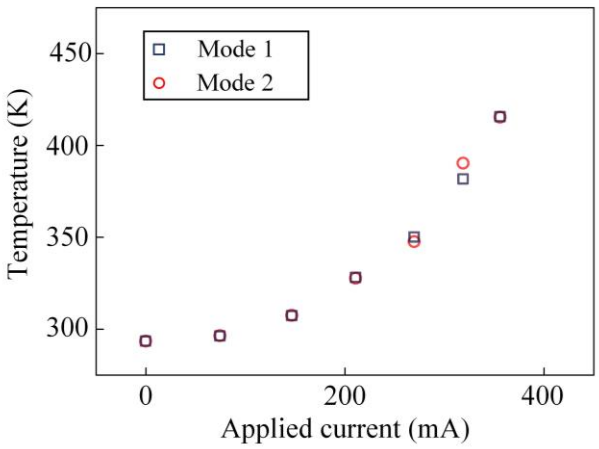

Supplementary Figure 8. Temperature variation in the thermal tuning process along with the increase of the applied current. Blue squares (mode 1) refer to the mode at 0.472 THz, while red circles (mode 2) represent the mode at 0.469 THz.

We note that the temperature change is proportional to the heating power related to Joule heat, and the Joule heat is squared to the applied current. The calculated results are shown in Supplementary Fig. 8. With the increase of the applied current, the temperature obeys a nearly square relationship. When the applied current increases from 0 mA to 356.4 mA, the temperature goes from 293.15 K up to around 414.9 K. Further increase the applied current, the temperature monotonically boosts.

S4.2 Isolation function at different central frequencies

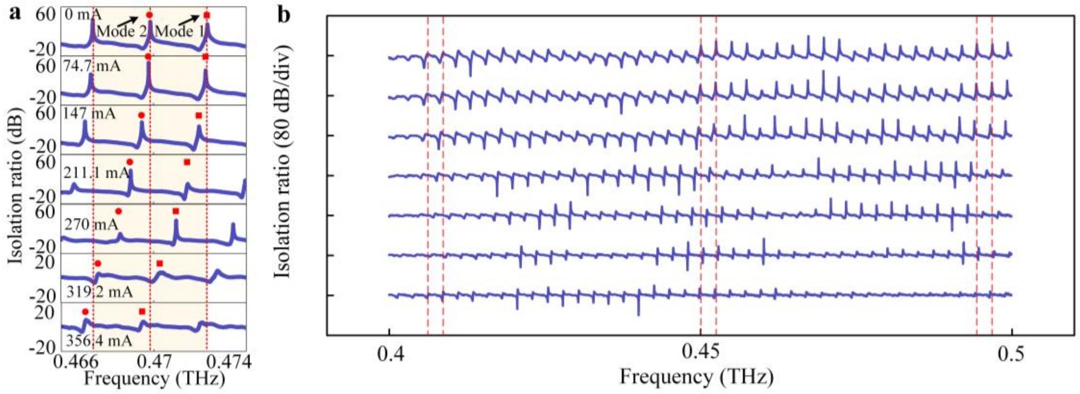

Supplementary Figure 9. a Isolation ratios of the chip in the change of the applied current when **B**=0.76 T. **b** Isolation ratios in the tuning process when **B**=0.76 T in a range of 0.405~0.495 THz. And the applied currents from up to bottom are 0, 74.7, 147, 211.1, 270, 319.2, and 356.4 mA, respectively.

When **B**=0.76 T, the isolation ratios of the chip in the complete tuning process are illustrated in Supplementary Fig. 9a. On the one hand, the central frequency of the isolation ratio can be adjusted over a frequency range of a single FSR. On the other

hand, the central frequency can be precisely controlled by applying different currents in the tuning process. Therefore, this isolator possesses great flexibility. Due to the tunability and the periodic resonances, we summarize that this function of isolation can be realized in a range of 0.405~0.495 THz. Supplementary Figure. 9b shows spectra of the isolation ratio in the entire frequency range during the tuning process. The isolation ratio can be observed in the whole frequency range and all modes can be tuned beyond a single FSR. Therefore, by optimizing the design, a great isolation effect with tunability can be achieved at the required frequency.

S4.3 Isolation ratios in the tuning process

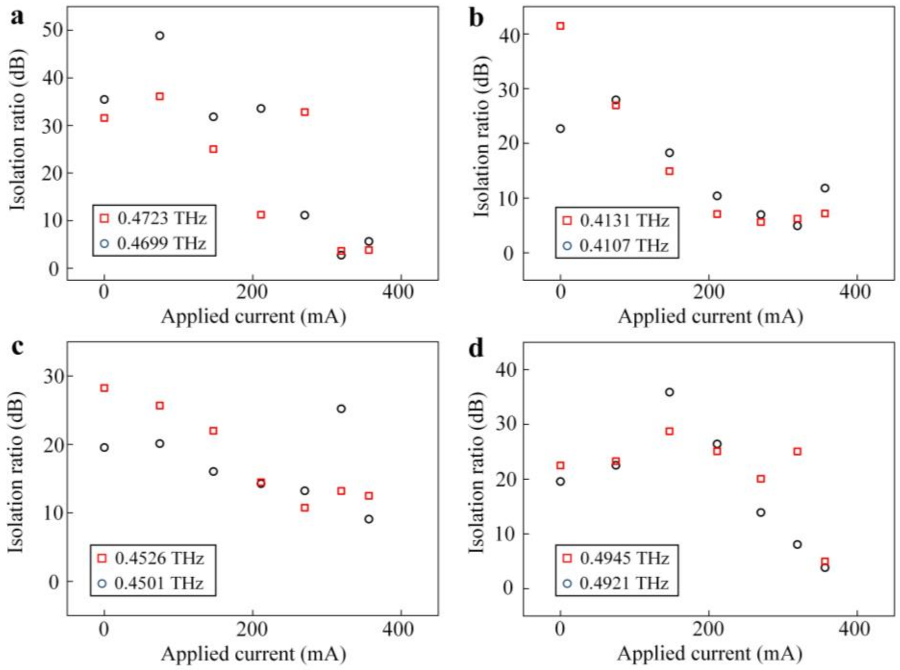

Supplementary Figure 10. Isolation ratios in the tuning process at different central frequencies related to Fig. 4 c and 4d.

In the thermal tuning process, we can observe the change of isolation ratios, as illustrated in Supplementary Fig. 10. The isolation ratios show a decreasing trend in general with the increase of the applied current. Meanwhile, increasing trends can be observed at several central frequencies. Based on the principle of the isolator, isolation ratios are determined by the joint interaction of the frequency detuning and the extinction ratio of the resonances. In the tuning process, the decrease of the frequency detuning and the extinction ratios will lead to reductions of the isolation ratios. Meanwhile, the increase of the extinction ratios can introduce increases in isolation ratios in certain situations.

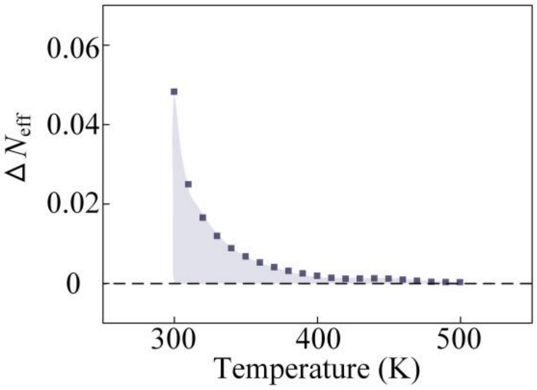

Supplementary Figure 11. The difference of the real part of the effective refractive indices in CW and CCW directions ($\Delta N_{\mathrm{eff}}=|N_{\mathrm{eff,CW}} - N_{\mathrm{eff,CCW}}|$) varies with the change of temperature when **B**=0.76 T, gap=8.76 μm.

First of all, the decreasing trend is the result of the decreasing frequency detuning. With the applied magnetic field, it can be observed that the frequency detuning decreases, as shown in Fig. 4a and 4b, which stems from the increase of the temperature

of the chip and the InSb.[7,8] The difference of effective refractive indices between CW and CCW directions ($|N_{\mathrm{eff,CW}}$-$N_{\mathrm{eff,CCW}}|$) with the increase of temperature is theoretically analyzed, as shown in Supplementary Fig. 11. When the temperature changes from 300 K to 500 K, we can find that the real part differences of the effective refractive indices gradually decrease from 0.05 to 0. Considering that a small deviation of effective refractive indices corresponds to a reduction in frequency detuning, it can be concluded that the experiment agrees with the theory.

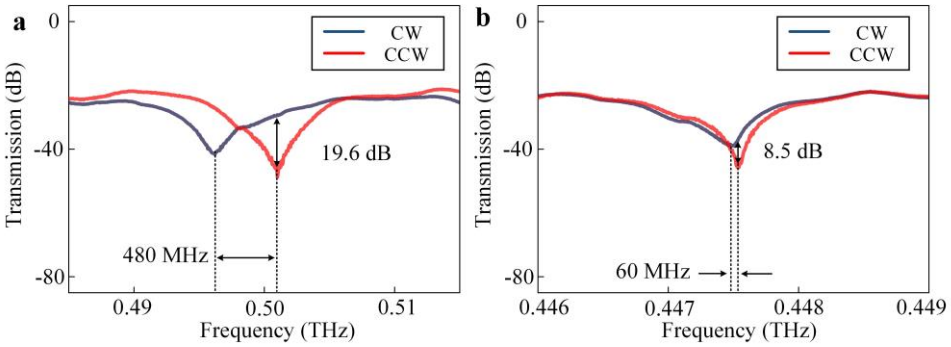

Supplementary Figure 12. Transmission spectra for **B**=0.76 T, when the applied currents are **a** 0 mA and **b** 356.4 mA, respectively.

A typical measured example of the influence for decreasing frequency detuning is illustrated in Supplementary Fig. 12. When there is no applied current, the frequency detuning is 480 MHz, and the related isolation ratio is 19.6 dB. While the applied current is set to 356.4 mA, the frequency detuning is 60 MHz, and the related isolation ratio is 8.5 dB. Considering that the CW extinction ratios are ~27 dB in both Supplementary Fig. 12a and 12b, the frequency detuning is the origin of the isolation ratio reduction.

Secondly, the increase of isolation ratios is the result of high extinction ratios at several conditions. For different temperatures, the coupling strengths (self-coupling coefficient (*r*)) and the loss of the resonator (the round-trip transmission coefficient (*a*)) will change. When the parameter *a* changes towards the condition *a*=*r*, the extinction ratio of the resonator increases. Considering that the parameter *a* in CW and CCW directions are different, when a high extinction ratio is realized in a single direction, we can obtain high isolation ratios.

A typical example is illustrated in Supplementary Fig. 13. Without applied currents, the extinction ratio of CCW mode is 27 dB, and the related isolation ratio is 19.6 dB. While the applied current is set to 319.2 mA, the extinction ratio of CCW mode is 39.2 dB, and the related isolation ratio is 25.2 dB. Considering that we can observe a significant decrease of frequency detuning, the extinction ratio is the origin of the isolation ratio increasing.

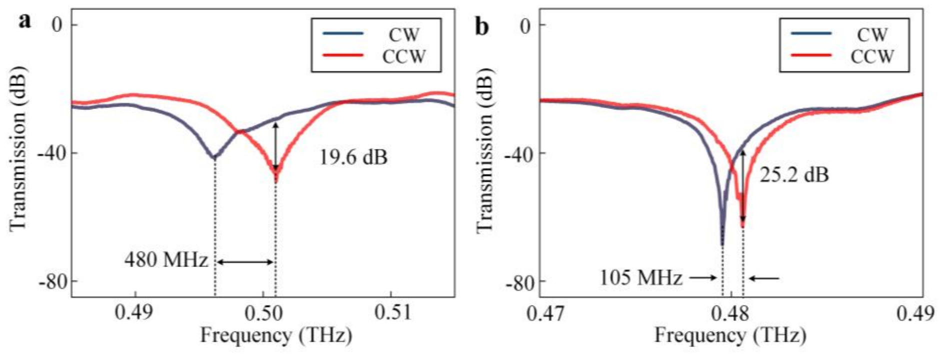

Supplementary Figure 13. Transmission spectra when **B**=0.76 T for the applied currents are **a** 0 mA and **b** 319.2 mA.

Supplementary References

1. Lin, S., Silva, S., Zhou, J., Talbayev, D. A One-Way Mirror: High-Performance Terahertz Optical Isolator Based on Magnetoplasmonics. *Adv. Opt. Mater.* **6**, 1800572 (2018).
2. Mu, Q.Y., *et al.* Tunable magneto-optical polarization device for terahertz waves based on InSb and its plasmonic structure. *Photonics Res.* **7**, 325-331 (2019).
3. Wang, X., Belyanin, A.A., Crooker, S.A., Mittleman, D.M., Kono, J. Interference-induced terahertz transparency in a semiconductor magneto-plasma. *Nat. Phys.* **6**, 126-130 (2009).
4. Fan, F., Chen, S., Chang, S.-J. A Review of Magneto-Optical Microstructure Devices at Terahertz Frequencies. *IEEE J. Sel. Top. Quantum Electron.* **23**, 1-1 (2016).
5. Yuan, S., Chen, L., Wang, Z., Wang, R., Wu, X., Zhang, X. Mode coupling in a terahertz multi-mode whispering-gallery-mode resonator. *Opt. Lett.* **44**, 2020-2023 (2019).
6. Wang, Z., Dong, G., Yuan, S., Chen, L., Wu, X., Zhang, X. Voltage-actuated thermally tunable on-chip terahertz filters based on a whispering gallery mode resonator. *Opt. Lett.* **44**, 4670-4673 (2019).
7. Vogt, D.W., Jones, A.H., Leonhardt, R. Thermal tuning of silicon terahertz whispering-gallery mode resonators. *Appl. Phys. Lett.* **113**, 011101 (2018).
8. Yuan, S., Chen, L., Wang, Z., Wang, R., Wu, X., Zhang, X. Tunable high-quality Fano resonance in coupled terahertz whispering-gallery-mode resonators. *Appl.*

Phys. Lett. **115**, 201102 (2019).
